# Genetic architecture of Alzheimer’s disease in a West African Cohort: Insights from the READD ‐ ADSP

**DOI:** 10.1002/alz.093308

**Published:** 2025-01-03

**Authors:** Rufus O. Akinyemi, Anthony J. Griswold, Motunrayo Coker, Patrice L. Whitehead, Farid Rajabli, Kazeem S. Akinwande, Samuel Diala, Mayowa Ogunronbi, Kyle Scott, Reginald Obiako, Larry D. Adams, Kara L. Hamilton‐Nelson, Kolawole Wahab, Pedro R. Mena, Albert Kwaku Akpalu, Brian W. Kunkle, Fred Stephen Sarfo, Jeffery M. Vance, Njideka U Okubadejo, Olusegun Baiyewu, Christiane Reitz, Giuseppe Tosto, Mayowa O Owolabi, William S. Bush, Jonathan L. Haines, Raj Kalaria, Goldie S. Byrd, Adesola Ogunniyi, Margaret A. Pericak‐Vance, African Dementia Consortium

**Affiliations:** ^1^ College of Medicine, University of Ibadan, Ibadan, Oyo State Nigeria; ^2^ Neuroscience and Aging Research Unit, Institute of Advanced Medical Research and Training, College of Medicine, University of Ibadan, Ibadan Nigeria; ^3^ John P. Hussman Institute for Human Genomics, University of Miami Miller School of Medicine, Miami, FL, USA, Miami, FL USA; ^4^ Dr. John T. Macdonald Foundation Department of Human Genetics, University of Miami Miller School of Medicine, Miami, FL USA; ^5^ University of Miami Miller School of Medicine, Miami, FL USA; ^6^ Institute for Advanced Medical Research and Training, College of Medicine, University of Ibadan, Ibadan, Oyo State Nigeria; ^7^ University of Miami, Miami, FL USA; ^8^ John P. Hussman Institute for Human Genomics, University of Miami Miller School of Medicine, Miami, FL USA; ^9^ Federal Medical Centre, Abeokuta, Ogun State Nigeria; ^10^ Ahmadu Bello University, Zaria, Kaduna Nigeria; ^11^ John P. Hussman Institute for Human Genomics, Miami, FL USA; ^12^ Univeristy of Ilorin Teaching Hospital, Ilorin, Kwara Nigeria; ^13^ University of Ghana, Accra Ghana; ^14^ John P. Hussman Institute for Human Genomics, Miller School of Medicine, University of Miami, Miami, FL USA; ^15^ Dr. John T. Macdonald Foundation Department of Human Genetics, Miller School of Medicine, University of Miami, Miami, FL USA; ^16^ Komfo Anokye Teaching Hospital, Kumasi Ghana; ^17^ African Dementia Consortium, Lagos Nigeria; ^18^ University College Hospital, Ibadan, Oyo State Nigeria; ^19^ G. H. Sergievsky Center, Vagelos College of Physicians and Surgeons, Columbia University, New York, NY USA; ^20^ Gertrude H. Sergievsky Center, Taub Institute for Research on the Aging Brain, Departments of Neurology, Psychiatry, and Epidemiology, College of Physicians and Surgeons, Columbia University, New York, NY USA; ^21^ Department of Population and Quantitative Health Sciences, Institute for Computational Biology, Case Western Reserve University, Cleveland, OH USA; ^22^ Newcastle University, Newcastle upon Tyne United Kingdom; ^23^ University of Nairobi, Nairobi Kenya; ^24^ Maya Angelou Center for Health Equity, Wake Forest University School of Medicine, Winston‐Salem, NC USA; ^25^ Wake Forest University School of Medicine, Winston‐Salem, NC USA

## Abstract

**Background:**

The “Recruitment and Retention for Alzheimer’s Disease Diversity Genetic Cohorts in the ADSP (READD‐ADSP)” is developing a resource to expand ancestral diversity in Alzheimer disease (AD) studies to dissect the genetic architecture of AD across different populations. In addition to US sites, READD‐ADSP includes four US sites and nine countries in sub‐Saharan Africa through the Africa Dementia Consortium (AfDC). The overall goal of READD‐ADSP is to identify genetically driven targets in diverse groups including African Americans and Hispanic/Latinos in US, and Africans. In this preliminary analysis we investigated the ancestral genetic differences and the impact of known AD risk factors within West African cohorts.

**Method:**

Genome‐wide genotyping was performed on 91 AD cases and 97 cognitive unimpaired controls from Nigeria and Ghana. *APOE* alleles and *ABCA7* deletion (rs142076058) were sequenced using Sanger. We calculated global ancestry (principal components) using the PC‐AiR approach that is robust to known and cryptic relatedness. We investigated known AD loci from non‐Hispanic White (NHW) and AA genome wide association studies. For association analysis, we employed a mixed‐model regression approach (SAIGE) where we controlled for age, gender, population substructure (first three principal components), and relatedness.

**Result:**

Principal component analysis identified a distinction between the Ghana and Nigerian cohorts along the first principal component (PC1). Among the genetic loci examined, several showed nominal significance. Notably, the most prominent marker was found in *SORL1* (rs17125523; p = 2 × 10^‐3^). Additionally, we discovered an exonic nonsynonymous marker in the BIN1 gene (rs112318500), which is specific to African ancestry and showed a protective effect. *APOE* e4 allele showed a significant association with AD risk (OR = 2.5; CI:1.5‐4.2; pv = 0.001), while the e2 indicated a protective trend but did not reach statistical significance. No statistical difference in the frequency of *ABCA7* deletion was observed between AD and CU individuals.

**Conclusion:**

Our findings highlight the presence of genetic variations between West African populations that warrant further investigation, potentially offering new insights into the genetic underpinnings of AD. Data collection is ongoing across the AfDC and updated data will be presented.

